# Detection of TDP-43 Proteinopathies in Brain and Cerebrospinal Fluid Using Seed Amplification Assay

**DOI:** 10.3390/cimb48080824

**Published:** 2026-08-13

**Authors:** Katsuya Satoh, Mika Inada Shimamura, Takeshi Fujimoto, Michio Kitayama, Akio Akagi, Yasushi Iwasaki, Yuu Satoh, Katsuhiro Ichinose, Akira Satoh, Ikuko Takahashi Iwata, Ichiro Yabe

**Affiliations:** 1 Unit of Medical and Dental Sciences, Department of Health Sciences, Graduate School of Biomedical Sciences, Nagasaki University, 1-7-7 Sakamoto, Nagasaki 852-8102, Japan; shima-m@nagasaki-u.ac.jp; 2 Department of Neurology, Sasebo City General Hospital, Sasebo 857-8511, Japan; fujimoto@hospital.sasebo.nagasaki.jp; 3 Department of Internal Medicine, Kawasaki Medical School, General Medical Center, Kurashiki 701-0192, Japan; mickitayama@med.kawasaki-m.ac.jp; 4 Department of Neuropathology, Institute for Medical Science of Aging, Aichi Medical University, Nagakute 480-1195, Japan; akio.akagi@gmail.com (A.A.); iwasakiyasushi1967@yahoo.co.jp (Y.I.); 5 Nagasaki-kita Hospital, Nagasaki 851-2103, Japan; y-satoh@shunkaikai.jp (Y.S.); kita_k_ichinose@shunkaikai.jp (K.I.); kita_a_satoh@shunkaikai.jp (A.S.); 6 Department of Neurology, Graduate School of Medicine, Hokkaido University, Sapporo 060-8638, Japan; ikukotak@med.hokudai.ac.jp (I.T.I.); yabe@med.hokudai.ac.jp (I.Y.)

**Keywords:** amyotrophic lateral sclerosis, frontotemporal lobar degeneration, TDP-43, biomarkers, seeding amplification assay

## Abstract

Misfolded TAR DNA-binding protein 43 (TDP-43) is the primary pathological hallmark of amyotrophic lateral sclerosis (ALS) and frontotemporal lobar degeneration (FTLD). While seed amplification assays (SAAs), such as real-time quaking-induced conversion (RT-QuIC), have shown promise in detecting misfolded TDP-43 in cerebrospinal fluid (CSF) and olfactory mucosa, technically accessible methodologies are urgently required for widespread clinical application. We developed a streamlined, non-immunoprecipitation-based TDP-43 RT-QuIC assay to assess seeding activity in brain tissue and CSF. We evaluated its diagnostic performance using CSF from patients with TDP-43 proteinopathies and control subjects, and further examined its association with neurofilament light chain (NfL) and tau-related biomarkers. In CSF analysis, the assay demonstrated positive seeding activity in 70% (21/30) of patients with ALS and dementia, 50% (5/10) of patients with FTLD, and 40% (8/20) of patients with ALS alone. The assay exhibited excellent specificity, yielding negative results in >99% (199/200) of control samples, including those with autoimmune or electrophysiological abnormalities. Furthermore, CSF analysis demonstrated significantly higher NfL levels in TDP-43 SAA-positive cases compared to SAA-negative cases (*p* < 0.0008). The highest NfL concentrations were observed in the SAA-positive ALS with dementia and ALS cohorts, contrasting with lower levels in FTLD. Tau-related biomarkers exhibited no significant differences between the groups. Our streamlined, non-immunoprecipitation TDP-43 RT-QuIC assay provides highly specific detection of pathological TDP-43 seeding activity. While the assay detects the underlying TDP-43 proteinopathy rather than distinguishing between ALS and FTLD clinical phenotypes, its technical simplicity and combined utility with NfL measurements offer a robust, scalable framework for biomarker development. This approach provides a practical foundation for future multi-center validation and international standardization efforts.

## 1. Introduction

Amyotrophic lateral sclerosis (ALS) is a fatal neurodegenerative disorder characterized by progressive loss of motor neurons, which are the nerve cells that control voluntary muscle movement. A key pathological hallmark of ALS is the accumulation of TAR DNA-binding protein 43 kDa (TDP-43) in the cytoplasm of neurons. TDP-43 is normally a nuclear protein that regulates critical cellular processes, including gene transcription, RNA splicing, transport of messenger RNA, and the formation of stress granules, which are protective structures that form under cellular stress [1]. Its essential role is underscored by the fact that complete loss of TDP-43 is lethal during embryonic development [1].

In 2006, TDP-43 was identified as the main component of abnormal protein inclusions in the brains and spinal cords of patients with ALS and frontotemporal lobar degeneration (FTLD), a related form of dementia [2]. TDP-43 pathology is found in approximately 97% of ALS cases and 45% of FTLD cases [3,4]. These observations highlight that ALS and FTLD are part of a spectrum of TDP-43 proteinopathies, sharing overlapping pathological mechanisms despite differing clinical presentations.

Importantly, misfolded TDP-43 aggregates can propagate between cells in a prion-like manner, meaning that abnormal proteins can induce normal proteins to adopt the same pathological structure [5,6,7,8]. This property has been exploited to develop sensitive detection methods called seed amplification assays (SAAs), including real-time quaking-induced conversion (RT-QuIC). In these assays, minute amounts of pathological TDP-43 “seeds” can be amplified in the laboratory and detected across various biological matrices, including cerebrospinal fluid (CSF) and olfactory mucosa [9,10]. While CSF and brain tissue provide direct access to pathological aggregates, measuring misfolded proteins in blood via SAAs is more challenging due to low peripheral concentrations and interference from other serum proteins, a limitation similarly documented in the development of blood-based alpha-synuclein SAAs [11,12].

Several studies have applied SAAs to detect misfolded TDP-43 in CSF [9,10]; however, reproducibility and standardization of these assays remain limited, preventing routine clinical use. In this study, we aimed to establish a simplified, non-immunoprecipitation-based RT-QuIC assay to detect pathological TDP-43 in brain and CSF samples from patients with sporadic ALS and FTLD. Rather than attempting to differentiate between ALS and FTLD clinical phenotypes, we sought to demonstrate the utility of this streamlined assay in identifying underlying TDP-43 proteinopathies and to evaluate its relationship with other established fluid biomarkers, such as neurofilament light chain (NfL).

## 2. Materials and Methods

### 2.1. Study Design

We aimed to establish and optimize a real-time quaking-induced conversion (RT-QuIC) assay to detect pathological TDP-43 seeding activity in brain tissue specimens, and subsequently apply this optimized assay to investigate seeding activity in CSF samples from patients with TDP-43 proteinopathies.

### 2.2. Patients

We utilized frozen postmortem brain tissue samples obtained from patients with pathologically confirmed TDP-43-positive FTLD and ALS. Sample type and handling were standardized for each analysis to ensure consistency. All patients or their legal representatives provided written informed consent for the use of autopsy materials in research. The study was conducted in accordance with the Declaration of Helsinki and the “Ethical Guidelines for Medical Research Involving Human Subjects” issued by the Ministry of Health, Labour and Welfare of Japan, and was approved by the Ethics Committee of the Nagasaki University Graduate School of Biomedical Sciences and Nagasaki University Hospital (ID No. UMIN000038398, UMIN000016855, and UMIN000003301).

Our retrospective study analyzed patients seen between 2017 and 2024 at Sasebo General Hospital and Nagasaki Kita Hospital. Clinical diagnoses of ALS and ALS with dementia were established based on the Gold Coast diagnostic criteria. Patients with TDP-43 FTLD met the criteria for Possible bvFTD in the 2011 revised edition by the International bvFTD Criteria Consortium (FTDC). We enrolled only patients who consented to participate in the study and agreed to undergo CSF collection. Informed consent was obtained from all participants or their legal guardians. This study utilized data stripped of all personal identifying information.

### 2.3. Brain Sample Preparation for TDP-43 RT-QuIC Assay

Frozen postmortem frontal lobe samples were homogenized (10% weight/volume) in phosphate-buffered saline (pH 7.0) and clarified by centrifugation at 6000× *g* for 5 min at 4 °C. The resulting supernatants were stored at −80 °C until analysis. Prior to the assay, the supernatants were serially diluted from 10^−6^ to 10^−9^ in phosphate-buffered saline. For each reaction well, 10 µL of the respective diluted homogenate was added to 90 µL of the reaction mixture to achieve a final reaction volume of 100 µL.

### 2.4. Expression and Purification of Recombinant TDP-43

Human TDP-43 constructs, including HuTDP-43 (1–414), TDP-35 (90–414), and TDP-25 (220–414), were cloned into the pET15b vector and expressed in *Escherichia coli* BL21(DE3) cells. Protein expression was induced with isopropyl β-D-1-thiogalactopyranoside (IPTG) under optimized conditions to maximize protein solubility.

Cells were harvested and lysed in an optimized lysis buffer, and the recombinant proteins were initially purified using SUMO-Trap FF chromatography. For preliminary screening assays requiring untagged proteins, the SUMO-tag was enzymatically cleaved using a SUMO-specific protease under proprietary conditions. The proteins were subsequently polished using size-exclusion chromatography and eluted into an assay-compatible buffer. Final protein purity was ≥99%, as assessed by sodium dodecyl sulfate polyacrylamide gel electrophoresis and mass spectrometry.

### 2.5. TDP-43 RT-QuIC Assay Optimization

All RT-QuIC reactions were performed in black, clear-bottomed 96-well plates (Nunc Company, Nunc 96 well; Sigma–Aldrich, St. Louis, MO, USA) with a 100 µL total reaction volume. To optimize amyloid seeding detection, we systematically tested multiple experimental parameters across various recombinant human TDP-43 constructs (with and without SUMO-tags) (Supplementary Table S1, Figures S1 and S2).

Based on this extensive optimization, the final reaction mixtures for brain samples contained phosphate-buffered saline or Tris-based buffers (pH 6.8–8.0) with NaCl (100 mM), thioflavin T (10–25 µM), sodium dodecyl sulfate (0.002%), and recombinant TDP-43 proteins (0.05–1 mg/mL). For each reaction, 10 µL of the serially diluted brain homogenate was added to 90 µL of the reaction mixture to achieve the final 100 µL volume. Plates were sealed and incubated at temperatures ranging from 40 °C to 55 °C with intermittent double-orbital shaking (400–1000 rpm, 1 min) and resting (1 min) cycles. Thioflavin T fluorescence was measured every 30 min using a FLUOstar OMEGA plate reader (BMG Labtech, Ortenberg, Germany) (excitation 450 nm, emission 480 nm).

### 2.6. CSF Analysis

CSF samples from 38 patients with TDP-43 proteinopathies and 200 controls were analyzed. For each reaction, 10 µL of neat CSF was added to 90 µL of the optimized reaction buffer (yielding a final reaction volume of 100 µL). The final reaction buffer contained 40 mM Tris (pH 8.0), 0.5 M guanidine hydrochloride, 10 µM thioflavin T, 0.002% sodium dodecyl sulfate, and 0.05 mg/mL of the optimal HuTDP-25 (220–414)-SUMO-tag substrate. Sealed plates were incubated at 40 °C for 50 h with double-orbital shaking (100 rpm for 15 s every 30 min).

### 2.7. Endpoint RT-QuIC Assay

We used the endpoint RT-QuIC method to quantitatively measure TDP-43 seeding activity within brain samples. The RT-QuIC reaction was performed as previously described. The Spearman–Kärber method was used to calculate the 50% seeding dose (*SD50*) in native brain tissue samples [13].

## 3. Results

### 3.1. TDP-43 Detection in Brain Samples

**Figure 1:**
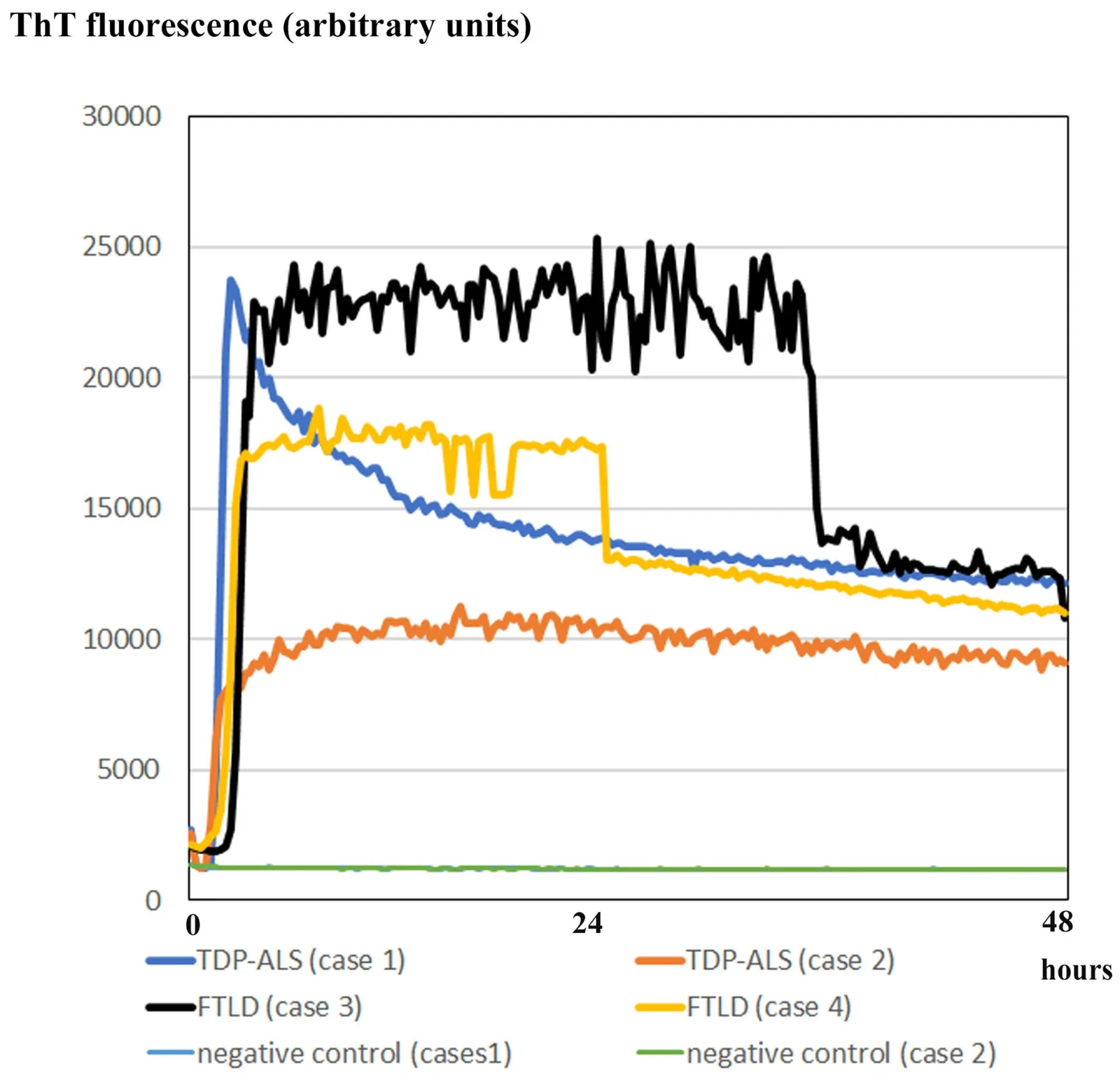
TDP-43 RT-QuIC kinetic profiles differentiate TDP-ALS and FTLD brain samples. Thioflavin T (ThT) fluorescence was monitored continuously over 48 h. TDP-ALS samples (cases 1 and 2) exhibited a rapid initial peak followed by a gradual decline, whereas FTLD samples (cases 3 and 4) showed a robust increase to a sustained high plateau. Negative controls (sudden death cases without pathology) maintained a stable baseline throughout the assay.

**Figure 2:**
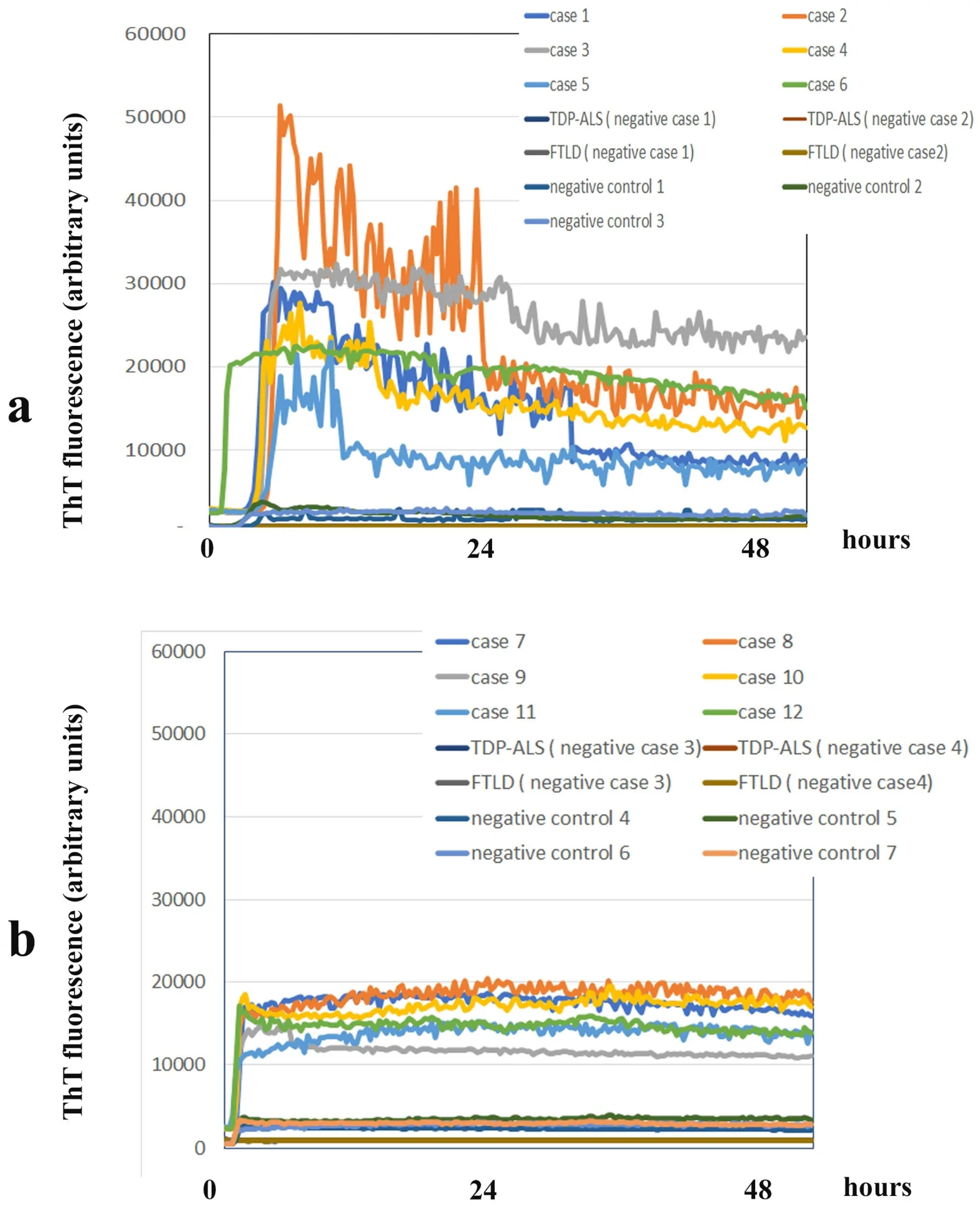
TDP-43 RT-QuIC kinetic profiles in CSF differentiate ALS and FTLD. Thioflavin T (ThT) fluorescence was monitored continuously over 48 h. (**a**) CSF samples from ALS patients (cases 1–3 with dementia; cases 4–6 with ALS alone) exhibit an immediate, rapid peak followed by a gradual decline, consistent with patterns observed in brain tissue. (**b**) CSF samples from FTLD patients (cases 7–12) demonstrate a rapid increase to a sustained fluorescence plateau. Across both panels, negative control CSF samples (representing individuals without TDP-43 pathology) maintain a stable, flat baseline, confirming the absence of abnormal seed amplification.

The RT-QuIC assay successfully detected TDP-43 seeding activity in brain homogenates from patients with TDP-43-positive FTLD and ALS. Representative kinetic curves illustrate the distinct amplification patterns (Figure 1). In samples from TDP-43-positive FTLD patients, Thioflavin T (ThT) fluorescence rapidly increased within 50 cycles and reached a sustained plateau (Figure 1). Interestingly, in brain samples from TDP-43-positive ALS patients, the assay exhibited an atypical kinetic profile characterized by an immediately high initial fluorescence baseline that gradually declined over time (Figure 1). Control brain samples maintained a low, flat baseline throughout the assay (Figure 1). Furthermore, analysis of the averaged kinetic profiles across the entire cohort confirmed the reproducibility of these distinct, disease-specific aggregation patterns (Figure 2).

The diagnostic criteria for sample positivity were established as follows. The fluorescence threshold was set at 100,000, and a well was considered positive if it crossed this threshold within 12 h. Each sample was analyzed in hexaplicate (6 wells per sample). A sample was defined as positive if at least 2 out of the 6 wells exhibited a positive signal. If a sample was initially negative (yielding 0 or 1 positive well), the assay was repeated. If the second run yielded 2 or more positive wells, the sample was ultimately classified as positive. In cases where exactly 1 well was positive in both the first and second runs, a third independent run was conducted. If this third run also resulted in exactly 1 positive well, the sample was definitively classified as negative.

We enrolled only patients who consented to participate in the study and agreed to undergo CSF collection. Informed consent was obtained from all participants or their legal guardians. This study utilized data stripped of all personal identifying information. Due to the retrospective nature of this study, systematic genetic screening (e.g., *C9orf72*, *TARDBP*, or *GRN* mutations) was not routinely performed. However, based on detailed clinical interviews regarding family history, all enrolled patients were clinically classified as having sporadic ALS or FTLD.

### 3.2. SD50 Analysis for TDP-43 Detection in Brain Samples

Table 1 shows the results of the log SD 50 analysis of brain homogenates from patients with TDP-43 proteinopathies using the RT-QuIC assay. We analyzed brain homogenates from four cases (Nos. 1–4; two TDP-43 cases and two FTLD cases). The log SD_50 values in brain homogenates were 7.91± 0.15 and 7.39 ± 0.83 for TDP-43 cases 1 and 2, respectively, and 6.93 ± 0.52 and 7.11 ± 0.73 for FTLD cases 3 and 4, respectively.

### 3.3. TDP-43 Detection in CSF Samples

Building upon the observations in brain tissue, the optimized RT-QuIC assay successfully detected pathological TDP-43 seeding activity directly from CSF. Crucially, the CSF analyses consistently recapitulated the distinct, disease-specific kinetic profiles originally identified in the brain homogenates across multiple independent biological replicates (Figure 2).

Representative amplification traces of CSF samples from the ALS spectrum are detailed in Figure 2a. This includes distinct, independent patients clinically diagnosed with ALS with dementia (Cases 1–3) and ALS alone (Cases 4–6). Irrespective of the presence of dementia, all TDP-43-positive ALS CSF samples exhibited the identical atypical kinetic profile observed in ALS brain tissues. This signature is characterized by a strikingly immediate and rapid increase in ThT fluorescence, reaching an initial peak within the first few hours of the assay, which is then predictably followed by a gradual, steady decline over the remainder of the 48-h monitoring period (Figure 2a).

In stark contrast, CSF samples obtained from distinct patients with TDP-43-positive FTLD (Figure 2b, Cases 7–12) demonstrated a fundamentally different amplification dynamic. These samples exhibited a robust, rapid sigmoidal increase in fluorescence that rapidly transitioned into a high, sustained plateau, maintaining a stable maximum fluorescence without the progressive decline characteristic of the ALS cohort. Across all corresponding runs, independent negative control CSF samples (Figure 2a,b; negative controls 1–7) maintained a perfectly stable, flat baseline throughout the entire 48 h, confirming the absence of spontaneous substrate aggregation.

Expanding this optimized non-immunoprecipitation TDP-43 RT-QuIC assay to our broader clinical cohort yielded robust diagnostic metrics (Table 2). The assay identified positive seeding reactions in 21 of 30 (70%) patients with ALS with dementia, 8 of 20 (40%) patients with ALS alone, and 5 of 10 (50%) patients with TDP-43 FTLD. Furthermore, the assay demonstrated exceptional analytical specificity; among the 200 independent control individuals, 199 (99.5%) yielded completely negative reactions. The single positive reaction in the control group was observed in a patient diagnosed with muscle-specific kinase (MuSK) antibody-positive myasthenia gravis. Together, these expansive results validate the high sensitivity and specificity of our streamlined approach for detecting central nervous system TDP-43 proteinopathy directly via CSF, effectively circumventing the well-documented technical hurdles of blood-based detection, such as critically low peripheral TDP-43 concentrations and severe serum protein interference.

### 3.4. CSF Neurofilament Light Chain and Total-Tau Protein Levels in ALS, ALS with Dementia, and FTLD Patients

**Figure 3:**
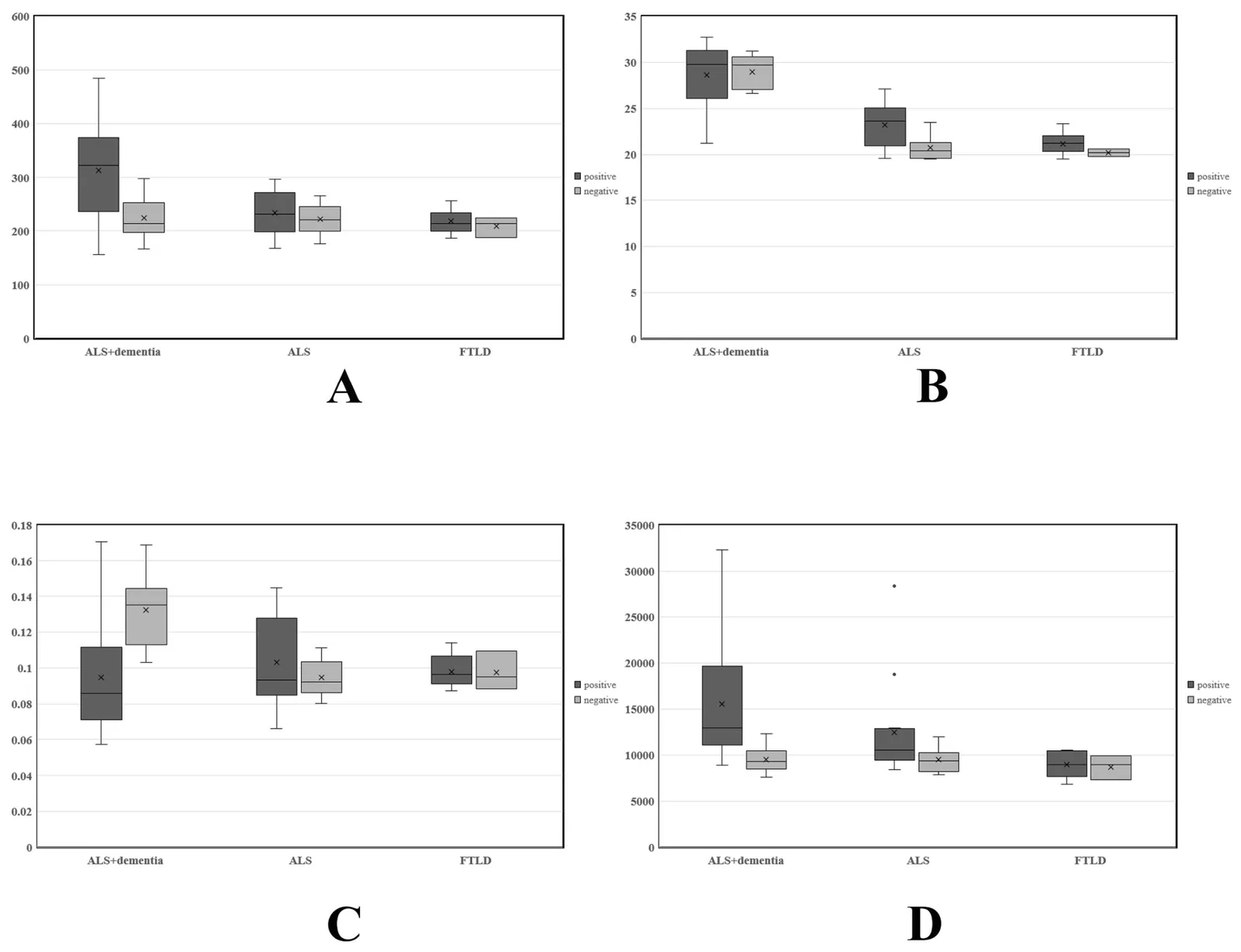
Comparison of CSF fluid biomarkers between TDP-43 RT-QuIC positive and negative cohorts. Box-and-whisker plots illustrating the levels of CSF biomarkers in patients clinically diagnosed with ALS with dementia, ALS alone, and FTLD. Patients are stratified by their TDP-43 seed amplification assay (SAA) results: SAA-positive (dark grey boxes) and SAA-negative (light grey boxes). (**A**) Total tau (t-Tau) levels (pg/mL). (**B**) Phosphorylated tau (p-Tau) levels (pg/mL). (**C**) The p-Tau/t-Tau ratio. (**D**) Neurofilament light chain (NfL) levels (pg/mL). The horizontal line within each box represents the median, the ‘×’ indicates the mean, and the lower and upper boundaries of the boxes represent the 25th and 75th percentiles, respectively. Whiskers extend to the minimum and maximum values excluding outliers, which are plotted as individual dots. Only CSF NfL levels (**D**) demonstrated a statistically significant elevation in the SAA-positive cohort compared to SAA-negative cases (*p* < 0.0008). No significant differences were observed in the tau-related biomarkers (**A**–**C**) between the groups.

To evaluate the clinical context of these seeding activities, we analyzed CSF levels of total tau (t-Tau), phosphorylated tau (p-Tau), the p-Tau/t-Tau ratio, and neurofilament light chain (NfL) between RT-QuIC positive (SAA-positive) and negative (SAA-negative) cases across the three clinical diagnostic groups (Table 1 and Figure 3).

Notably, only CSF NfL levels demonstrated a statistically significant elevation in the SAA-positive cohort compared to the SAA-negative cases (*p* < 0.0008). Within the specific clinical subgroups, the highest NfL levels were observed in the SAA-positive ALS with dementia group (mean ± SD: 15,497.76 ± 5945.34 pg/mL), followed by the SAA-positive ALS group (12,472.46 ± 5453.81 pg/mL). In contrast, SAA-positive FTLD cases exhibited considerably lower NfL levels (8964.00 ± 1432.32 pg/mL), which were comparable to those of the SAA-negative groups (ALS with dementia: 9530.89 ± 1440.98 pg/mL; ALS: 9487.00 ± 1366.57 pg/mL; FTLD: 8717.67 ± 1313.40 pg/mL).

Conversely, there were no significant differences in tau-related biomarkers between the SAA-positive and SAA-negative groups. In the ALS with dementia cohort, t-Tau levels were 313.67 ± 83.68 pg/mL in SAA-positive and 224.33 ± 39.96 pg/mL in SAA-negative cases. Similarly, p-Tau levels (28.58 ± 3.36 vs. 28.98 ± 1.80 pg/mL) and the p-Tau/t-Tau ratio (0.095 ± 0.033 vs. 0.132 ± 0.021) did not show significant variations driven by SAA positivity. This lack of significant difference in tau profiles was consistent across the ALS (SAA-positive t-Tau: 233.77 ± 42.96 pg/mL; p-Tau: 23.22 ± 2.34 pg/mL) and FTLD (SAA-positive t-Tau: 217.71 ± 22.61 pg/mL; p-Tau: 21.17 ± 1.23 pg/mL) groups when compared to their respective SAA-negative counterparts (Table 3 and Figure 3).

## 4. Discussion

In the present investigation, we examined the seeding potential of various recombinant proteins for detecting TDP-43 activity, with a primary focus on establishing a reliable assay for cerebrospinal fluid (CSF) samples. Our analysis successfully identified pathological TDP-43 seeding activity using a streamlined RT-QuIC assay.

We hypothesized that an effective TDP-43 recombinant protein must incorporate the prion-like domain across residues 274–414. Initially, we explored the development of an untagged recombinant protein. However, full-length and untagged TDP-43 inherently possess a severe tendency to spontaneously aggregate. This intrinsic aggregation makes it exceptionally difficult to obtain a pure, stable substrate without a solubility tag, a challenge that aligns with the broader consensus among TDP-43 researchers. To overcome this critical hurdle, we utilized a SUMO-tag to maintain protein solubility during expression and purification. Through extensive screening, we determined that the HuTDP-25 (220–414)-SUMO-tag construct provided exceptional stability and high yield. Crucially, retaining the SUMO-tag did not hinder the conformational conversion process; rather, this specific TDP-25-SUMO-tag substrate exhibited robust, highly reproducible seeding activity. Therefore, we established the TDP-25-SUMO-tag as the definitive primary substrate for our streamlined RT-QuIC platform, which proved particularly effective for detecting minute seeds directly from CSF.

The TDP-43 seed amplification (RT-QuIC) assay has been previously described; however, achieving reproducible results across independent laboratories has proven exceptionally challenging. Compared with conventional biochemical or immunoassay-based detection methods (such as ELISA or western blot), RT-QuIC offers distinct advantages by exploiting the self-propagating nature of misfolded proteins. This technique amplifies minute amounts of pathological “seeds” through cycles of incubation and shaking, detected in real time by thioflavin-T fluorescence. Unlike static assays, RT-QuIC directly measures pathogenic seeding activity, thereby achieving high analytical sensitivity and specificity even in samples with extremely low aggregate abundance.

Recent investigations have established the feasibility of detecting TDP-43 seeding activity across diverse biological matrices, including CSF [14,15,16,17,18] and olfactory mucosa [9,10], alongside the development of enhanced sensitivity platforms [19,20,21,22]. Although these advances validate the detection of pathological aggregates, a critical unmet need persists: the development of technically accessible, reproducible methodologies suitable for widespread clinical implementation. Our streamlined approach directly addresses this translational gap. While immunoprecipitation can enhance analytical performance, it substantially increases assay costs and introduces variability due to lot-to-lot antibody inconsistency, making it difficult to standardize in routine clinical laboratories. By contrast, our method minimizes batch-to-batch differences, highlighting its potential as a highly scalable option.

Sample source selection also constitutes a pivotal consideration. While olfactory mucosa sampling [9,10] represents an important step toward minimally invasive diagnostics, our strategic focus on CSF confers distinct clinical advantages [14,15,16,17]. Lumbar puncture is an established component of standard neurological practice, and the robust detection of TDP-43 seeding activity in CSF provides direct access to central nervous system pathology without the interference commonly encountered in blood-based assays [19,20,21,22].

One of the most intriguing observations of our study is the distinct fluorescence amplification kinetics between ALS and FTLD samples. We hypothesize that the biological basis for these divergent amplification profiles is rooted in disease-specific structural conformations, or “strains,” of the TDP-43 aggregates. This phenomenon closely parallels the behavior observed in alpha-synuclein RT-QuIC assays, where Lewy body dementia (LBD) and multiple system atrophy (MSA) exhibit distinct kinetic signatures driven by different strain conformations. Our kinetic data strongly suggest that the differences in TDP-43 seeding behavior between FTLD and ALS are similarly determined by fibril polymorphism. Furthermore, this hypothesis is heavily supported by recent Cryo-EM studies, which have revealed that TDP-43 aggregates in FTLD and ALS are structurally distinct.

Our findings demonstrate that the current TDP-43 SAA effectively detects underlying TDP-43 pathology. Currently, both ALS and FTLD exhibit overlapping seeding activities, meaning the assay functions primarily as a broad, highly specific marker of TDP-43 proteinopathy rather than a standalone tool to differentiate the two clinical phenotypes. To evaluate the clinical context, we analyzed concurrent fluid biomarkers. CSF NfL levels were significantly elevated in the SAA-positive cohort (*p* < 0.0008). Pathophysiologically, this elevation likely reflects the massive axonal efflux driven by the pervasive degeneration of motor axons inherent to ALS and ALS with dementia, which contrasts with the predominantly cortical involvement of pure FTLD.

We acknowledge a limitation in our study regarding the statistical analysis of these fluid biomarkers. We did not perform multivariate correction for known covariates, such as age and sex, when comparing NfL levels across the groups. However, we consider that the profound pathological surge in NfL driven by disease-induced axonal destruction far outweighs any physiological variations related to aging or sex. Therefore, it is highly unlikely that demographic imbalances would alter our primary conclusion regarding the notably high NfL levels in the SAA-positive ALS cohorts. Nonetheless, future studies utilizing larger, demographically matched cohorts are warranted to validate these findings using multivariate analyses.

Additionally, we acknowledge that the overall sample size (60 cases) is relatively small. However, this represents the entirety of our currently available, rigorously characterized dataset. We present this study as an important initial foundational step. Furthermore, regarding detailed clinical information such as total disease duration, we cannot provide this for all patients because the cohort consists of retrospectively collected samples where such data was not uniformly recorded. Additionally, we have reviewed recent literature and confirmed that it is common practice in many studies focusing on sporadic cases not to perform comprehensive genetic testing. Therefore, the absence of this specific data in our cohort aligns with the current accepted practices in similar exploratory studies.

Although our current study focuses on the diagnostic utility of our RT-QuIC assay in CSF and brain tissues, we acknowledge the critical need for further methodological optimization to enhance sensitivity and inter-laboratory reproducibility. Recently, novel methodological advancements, such as the micelle-assisted seed amplification assay described by Sakamoto et al., have shown great promise in achieving ultrasensitive detection of TDP-43 aggregates [18].

Furthermore, while CSF remains a primary matrix for evaluating central nervous system proteinopathies, there is a rapidly growing field exploring TDP-43 seeding activity in other, less invasive biofluids and peripheral tissues. For instance, recent studies have successfully detected TDP-43 seeds in the olfactory mucosa of patients with ALS [16]. The clinical utility of these assays is also expanding; TDP-43 seeds have now been detected even in the CSF of presymptomatic and symptomatic patients with genetic FTD/ALS [17]. Coupled with advanced quantitative cell-based reporters that directly link TDP-43 aggregation to pathogenic dysfunction [10]., these ultrasensitive assays will not only improve early and non-invasive diagnostics but also deepen our understanding of disease pathogenesis.

Although our current attempts to develop a definitive differential scoring system incorporating NfL levels and disease stage did not yield clinically robust indicators, this represents a highly promising frontier for future research. If future methodological refinements—such as identifying strain-specific kinetic signatures, distinct substrate cleavage profiles, or integrating novel multiplexed biomarkers—enable the clear differentiation between ALS and FTLD, this CSF-based platform will evolve into an exceptionally powerful tool. The ability to distinguish these phenotypes biochemically would revolutionize early diagnosis, prognosis, and the precise stratification of patients for targeted clinical trials.

Furthermore, expanding the application of this assay to aging populations holds immense promise for the in vivo detection of limbic-predominant age-related TDP-43 encephalopathy (LATE). Given the clinical overlap between LATE and other neurodegenerative dementias, the ability to identify LATE-specific TDP-43 seeding signatures would address a critical diagnostic blind spot, ultimately maximizing the clinical and therapeutic utility of this biomarker platform.

## 5. Conclusions

The present study demonstrates the successful detection of misfolded TDP-43 aggregates in CSF and brain samples using a streamlined RT-QuIC assay. optimized with the TDP-25-SUMO-tag substrate. These findings provide a strong proof-of-concept for the clinical application of CSF-based TDP-43 seed amplification assays. While the current assay robustly confirms the presence of broad TDP-43 proteinopathy, future advancements that enhance the ability to distinguish specific disease phenotypes will ultimately maximize the clinical and therapeutic utility of this innovative biomarker platform.

## Figures and Tables

**Table 1:** SD50 analysis of amyloid seeding activities in bain analysis of TDP-43 proteinopathy patients.

	log SD50 in Brain Homogenates
TDP-43 case 1	7.91 ± 0.15
TDP-43 case 2	7.39 ± 0.83
FTLD case 3	6.93 ± 0.52
FTLD case 4	7.11 ± 0.73

**Table 2:** Positive rate of amyloid seeding activities in CSF analysis of TDP-43 proteinopathy patients.

	ALS with Dementia	ALS	FTLD	Negative Controls
Number (N)	30	20	10	199
Age at onset (years old)	65.0 ± 12.9	52.0 ± 9.4	53.5 ± 7.1	72.3 ± 12.2
Sex male:female	7:3	13:7	7:3	5.9:4.1
Positive rate of TDP-43 seeding activities	70%	40%	50%	0.50%

**Table 3:** CSF biomarkers by TDP-43 Seeding Activity among CSF samples of TDP-43 proteinopathy patients.

	ALS + Dementia		ALS		FTLD	
TDP-43 seed amplification assay	positive	negative	positive	negative	positive	negative
total tau protein (pg/mL)	313.7 ± 83.7	224.3 ± 40.0	233.8 ± 43.0	221.9 ± 29.7	217.7 ± 22.6	208.3 ± 18.4
phosphorylated -tau protein (pg/mL)	28.6 ± 3.4	29.0 ± 1.8	23.2 ± 2.3	20.7 ± 1.4	21.2 ± 1.2	20.2 ± 0.4
phosphorylated tau/total tau ratio	0.1 ± 0.0	0.1 ± 0.0	0.1 ± 0.0	0.1 ± 0.0	0.1 ± 0.0	0.1 ± 0.0
Neurofilament light chain (pg/mL)	15,497.8 ± 5945.3	9530.9 ± 1441.0	12,472.5 ± 5453.8	9487.0 ± 1366.6	8964.0 ± 1432.3	8717.7 ± 1313.4

## Data Availability

All data and code used in this study will be made publicly available upon acceptance of the manuscript for publication.

## Supplementary Materials

The following supporting information can be downloaded at: https://www.mdpi.com/article/10.3390/cimb48080824/s1.
