# Supplementary material for: Detection of TDP-43 Proteinopathies in Brain and Cerebrospinal Fluid Using Seed Amplification Assay
Source: Curr Issues Mol Biol. 2026 Aug 13;48(8):824. doi: 10.3390/cimb48080824 (PMC13510334; doi:10.3390/cimb48080824)
Supplement: Supplementary file 1 [file cimb-48-00824-s001.zip › cimb-4431593-supplementary.pdf]

Supplementary Appendix

This appendix provides readers with additional information about this study.

Table of Contents

Authors and their contributions P1

Supplementary Figure S1: Purification of recombinant TDP-25- SUMO-tag protein P2

Supplementary Table S1: List of assays for all first recombinant protein P3

Supplementary Figure S2: Analysis of TDP-43 QuIC assay in brain homogenate samples from patients with TDP-43-positive frontotemporal lobe dementia (FTLD) and amyotrophic lateral sclerosis (ALS) using five TDP-43 recombinant proteins P4

Authors and their contributions

Katsuya Satoh, MD,[1]* Takeshi Fujimoto, MD,[2] Michio Kitayama, MD,[3] Akio Akagi, M.D.,[4] Yasushi Iwasaki, MD,[4] Yuu Satoh, MD,[5] Katsuhiro Ichinose, MD,[5] Akira Satoh, MD,[5] Ikuko Takahashi Iwata,[6] Ichiro Yabe,[6]

[1]Department of Health Sciences, Unit of Medical and Dental Sciences, Nagasaki University Graduate School of Biomedical Sciences, Nagasaki City, Nagasaki Prefecture, Japan.
[2]Department of Neurology, Sasebo City General Hospital, Sasebo, Nagasaki Prefecture, Japan.
[3]Department of Internal Medicine Kawasaki Medical School General Medical Center, Kurashiki, Okayama Prefecture, Japan
[4]Department of Neuropathology, Institute for Medical Science of Aging, Aichi Medical University, Aichi, Aichi Prefecture, Japan.
[5]Nagasaki-kita Hospital, Tokitsu, Nagasaki Prefecture, Japan.
[6]Department of Neurology, Graduate School of Medicine, Hokkaido University, Sapporo City, Hokkaido Prefecture, Japan

Authors' contributions

Manuscript writing: KS. Neuropathological analysis: AA and YI. CSF sample collection and participation enrollment: KS, TK, MK, YS, KI, AS, II, and IY. All authors provided feedback on the manuscript. All authors read and consented to the publication of the final version of the manuscript.

.

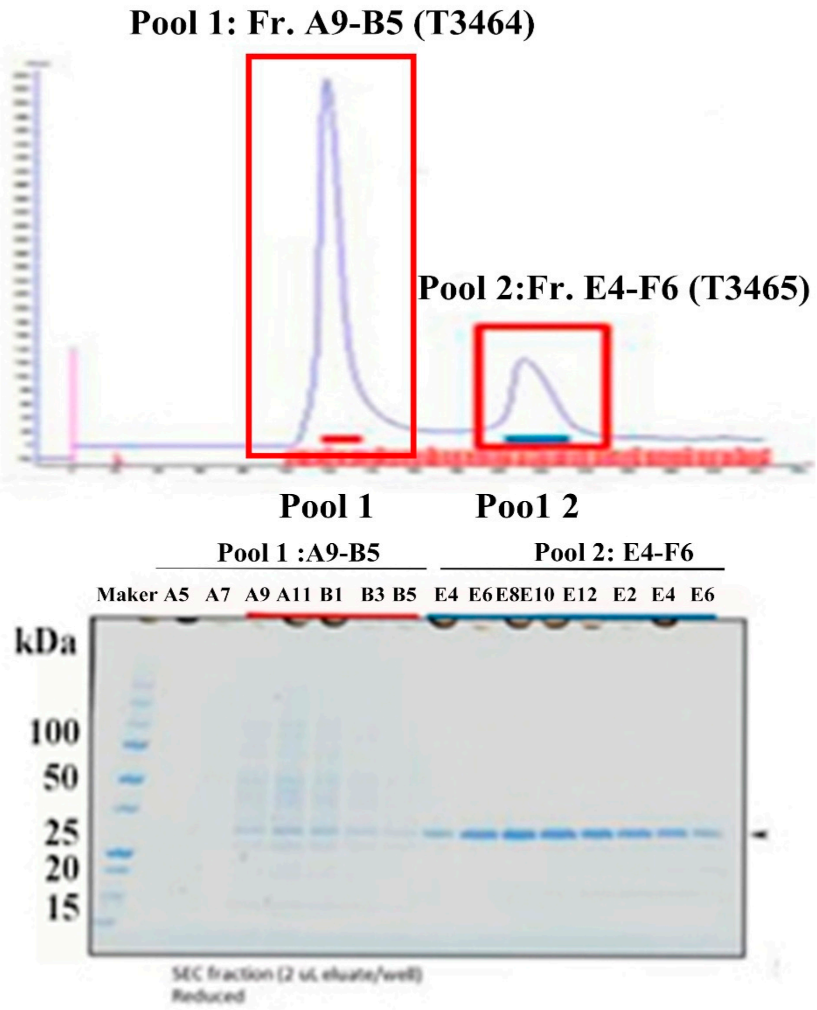

Supplementary Figure 1

Supplementary Figure S1: Purification of recombinant TDP-25- SUMO-tag protein

(A) *HPLC purification profile of the recombinant TDP-25-SUMO-tag protein.*

Fractions corresponding to Pool 1 (A9–B5) and Pool 2 (E6–F6) are indicated by colored bars.

Pool 2 contains the major peak within this elution window.

(B) *SDS–PAGE analysis of selected fractions from Pool 1 and Pool 2.*

Fractions A9, A11, B1, B3, and B5 (Pool 1) and E4, E6, E8, E10, and E12 (Pool 2) were analyzed under reducing conditions.

A prominent band corresponding to the recombinant TDP-25-SUMO-tag protein (~25 kDa, indicated by arrow) was detected predominantly in fractions E8 and E10, consistent with >95% purity.

Fractions from Pool 1 contained only minimal or undetectable amounts of the target protein.

These results confirm that the high-purity recombinant substrate used for the RT-QuIC assay was obtained specifically from a narrow elution range within Pool 2.

Supplementary Table S1. Assays for the first recombinant proteins

This table provides a comprehensive list of assays conducted with various recombinant proteins, specifically those utilized in the TDP-43 QuIC assay.

TDP	Concentration (mg/mL)	Concentration of Recomobinat protein (µg/ml)	Temperature (°C)	shaking speed (rpm)
TDP-43-sumo	0.17	100,110,120	50	800, 900, 1,000
	0.11	60,70,80	55	800
TDP-35-sumo	0.074	50	55	800
TDP-25-sumo	0.39	100,110,120	40,45,50,55	800, 900, 1,000
	0.14	100	55	800
	0.13	90	55	800
TDP-25	0.073	50	55	800
	0.051	35	55	800

Supplementary Figure S2: TDP-43 RT-QuIC assay analysis of brain homogenates from patients with FTLD and ALS using different recombinant proteins.

We performed TDP-43 RT-QuIC assays using five recombinant proteins, as detailed in Supplementary Table 1. Note that the TDP-25-sumo-tag recombinant protein was excluded from this analysis because it exhibited no seeding activity against brain tissues from either TDP-43-positive FTLD or ALS patients.

The panels are organized by sample type and the specific recombinant protein used:

- Panels A–E: Negative controls
- Panels F–J: Brain tissues from TDP-43-positive FTLD patients
- Panels K–O: Brain tissues from ALS patients

Across these sample groups, the specific recombinant proteins are labeled as follows:

- A, F, and K: TDP-43 recombinant protein
- B, G, and L: TDP-35 recombinant protein
- C, H, and M: TDP-25 recombinant protein
- D, I, and N: TDP-43-sumo-tag recombinant protein
- E, J, and O: TDP-35-sumo-tag recombinant protein

The y-axis represents ThT fluorescence intensity in arbitrary units (A.U.), and the x-axis represents the number of assay cycles. Although a positive response across four replicate wells was observed in one singular case, no statistically significant non-specific amplification was detected overall.

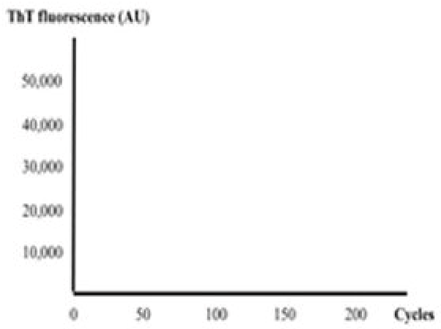

	TDP-43	TDP-35	TDP-25	TDP-43-SUMO	TDP-35-SUMO
Negative control	A	B	C	D	E
TDP-43 positive FTLD (Patient)	F	G	H	I	J
TDP-43 positive ALS (Patient)	K	L	M	N	O

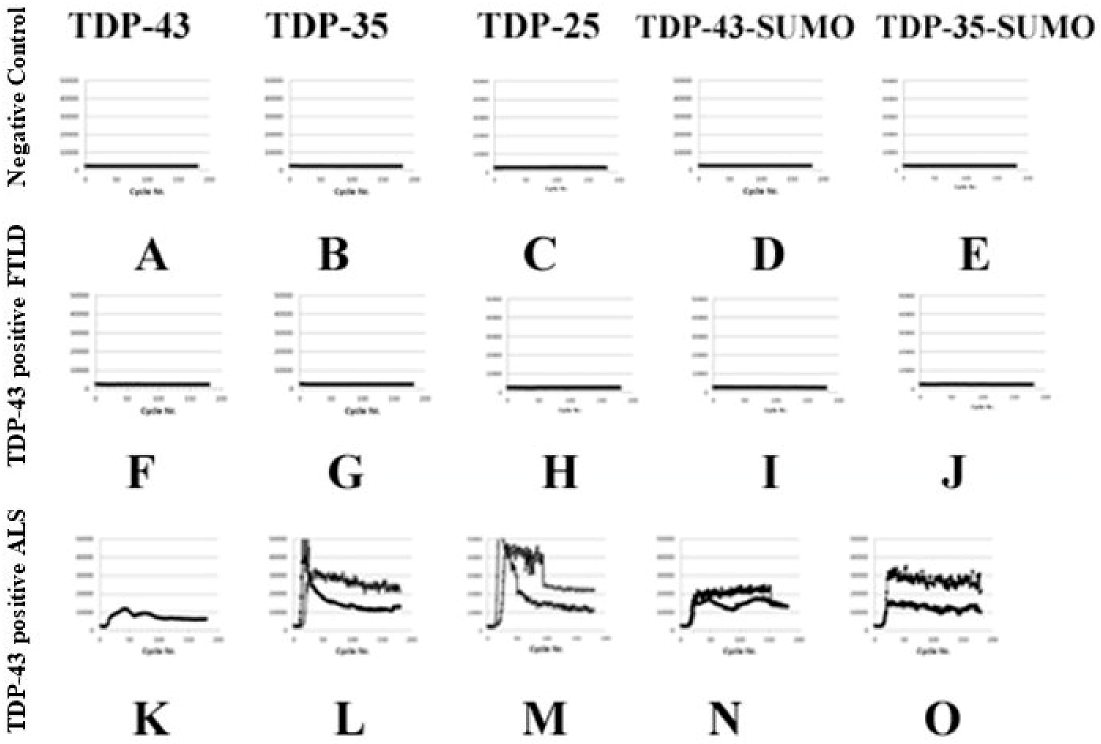

Supplementary Figure 2
